# Unlocking the undruggable spliceosome: generative AI and structural dynamics in cancer therapy

**DOI:** 10.3389/fcell.2026.1774239

**Published:** 2026-02-27

**Authors:** Jakob Steuer, Abdullah Kahraman

**Affiliations:** 1 Data Science in Life Sciences Group, Institute for Chemistry and Bioanalytics, School of Life Sciences, FHNW University of Applied Sciences and Arts Northwestern Switzerland, Muttenz, Switzerland; 2 Swiss Institute of Bioinformatics, Amphipôle, Quartier UNIL-Sorge, Lausanne, Switzerland

**Keywords:** biomarker, cancer, generative AI, molecular dynamics, neoantigens, spliceosome

## Abstract

The spliceosome is a dynamic molecular machine essential for transcriptome diversity, yet its complexity creates specific vulnerabilities in cancer. Recurrent somatic mutations in core factors, particularly SF3B1, U2AF1, and SRSF2, drive malignancies by altering splice-site recognition. Such structural perturbations do not merely drive oncogenesis but manifest as distinctive molecular signatures that can serve as potent diagnostic and prognostic biomarkers. However, therapeutic exploitation of these defects remains challenging. This review argues that unlocking the spliceosome requires a shift from static cryo-EM snapshots to dynamic structural ensembles. We explore how physics-based molecular simulation and enhanced sampling methods are merging with generative Artificial Intelligence to identify intermediate states, map cryptic allosteric pockets and target intrinsically disordered regions. Translating these mechanistic insights into the clinic, we evaluate the next-generation of therapeutic strategies, ranging from novel molecular biomarkers to rationally designed allosteric modulators and synthetic lethality. Finally, we discuss how deciphering these altered structural dynamics can guide the identification of splicing-derived neoantigens and biomarkers, establishing a roadmap for precision immunotherapy.

## 1 Introduction

The faithful expression of the eukaryotic genome is dependent on the spliceosome, a megadalton ribonucleoprotein (RNP) complex that orchestrates the removal of introns and the ligation of exons from precursor messenger RNA (pre-mRNA). This process is not merely a housekeeping function, but rather a fundamental engine of proteomic diversity. Through alternative splicing, a limited number of genes generate a vast repertoire of protein isoforms with distinct functions. These processes drive cellular differentiation, tissue homeostasis, and organismal complexity (Chen and Manley, 2009; Wang et al., 2015). In humans, more than 95% of multi-exon genes are reported to undergo alternative splicing, a flexibility that is essential for life but also represents a critical vulnerability (Lu et al., 2012; Jiang and Chen, 2021). The spliceosome, a complex intron-retaining ribonucleoprotein, is made up of five small nuclear RNAs (snRNAs) and hundreds of proteins. This intricate assembly necessitates a highly coordinated process of assembly and disassembly on each pre-mRNA substrate.

Within the context of cancer, this machinery is frequently co-opted. Somatic mutations in genes encoding core splicing factors, most notably *SF3B1, U2AF1*, and *SRSF2*, have emerged as cardinal drivers of hematologic malignancies such as myelodysplastic syndromes (MDS) (Yoshida et al., 2011) and chronic lymphocytic leukemia (CLL) (Quesada et al., 2012), as well as solid tumors like uveal melanoma and breast cancer (Harbour et al., 2013; Ellis et al., 2012). In contrast to typical loss-of-function mutations observed in tumor suppressor genes, these are heterozygous, change-of-function mutations that fundamentally alter the structural dynamics of spliceosome assembly (Zhang et al., 2021). The process does not result in the destruction of the splicing machinery. Instead, it involves the reprogramming of its thermodynamic preferences, thereby redirecting it to recognize aberrant splice sites (Zhang et al., 2024). This generates tumor-specific isoforms that can drive oncogenesis, confer drug resistance, or create potentially immunogenic neoantigens. Integrating structural dynamics with large-scale transcriptomics (multi-omics) allows for the identification of isoform-level biomarkers. These signatures can stratify patients into risk groups, serving as prognostic indicators for disease progression in MDS and CLL (Tseng and Obeng, 2024; Qiu et al., 2016; Zhang et al., 2017).

For decades, the spliceosome was considered an “undruggable” target because of its sheer size, lack of deep hydrophobic pockets in its static states, selectivity challenges and extraordinary dynamic nature. The splicing cycle involves a continuous, ATP-driven remodeling of the U1, U2, U4, U5, and U6 snRNPs (Wahl et al., 2009). While the “resolution revolution” in cryogenic electron microscopy (cryo-EM) has provided atomic-level maps of the spliceosome’s discrete conformational states, these structures historically represent snapshots of deep energy minima (Fica and Nagai, 2017; Tholen and Galej, 2022). They often fail to capture the “breathing” motions, intrinsic disorder, and transient transition states where therapeutic opportunities frequently lie. A convergence of technological breakthroughs is reshaping this landscape. The integration of computational biophysics, particularly Molecular Dynamics (MD) simulations, with generative Artificial Intelligence (AI) is enabling a shift from static structural snapshots to rich dynamic ensembles. These approaches make it possible to characterize the fluctuations of splicing factors, reveal transient “cryptic” allosteric pockets (Vajda et al., 2018) exposed during the splicing cycle, and computationally design small molecules and *de novo* protein binders that engage intrinsically disordered regions (IDRs), which have traditionally been deemed intractable (Sun et al., 2025).

The mutational landscape (Zhang et al., 2024), cryo-EM based structural foundations (Vorländer et al., 2022; Tholen and Galej, 2022), and clinical potential (Eymin, 2021; Anczukow et al., 2024; Lv et al., 2025) of the spliceosome have been extensively reviewed. More recently, the field has begun to appreciate the machinery’s fluid nature, and how molecular simulations complement experimental data to decode spliceosome dynamics (Borišek et al., 2023; 2021; Pokorná et al., 2025). This review builds upon these mechanistic foundations but pivots toward the therapeutic application of these dynamics using emerging technologies. We move beyond the characterization of equilibrium fluctuations to explore how Generative AI and biomolecular language models are reshaping the design of binders for transient pockets and intrinsically disordered regions. Finally, we extend this structural perspective into the clinical realm, linking dynamic conformational defects directly to synthetic lethal vulnerabilities and the systematic discovery of splicing-derived neoantigens, proposing a forward-looking roadmap for next-generation precision medicines.

## 2 Dynamics of the canonical splicing cycle

**FIGURE 1:**
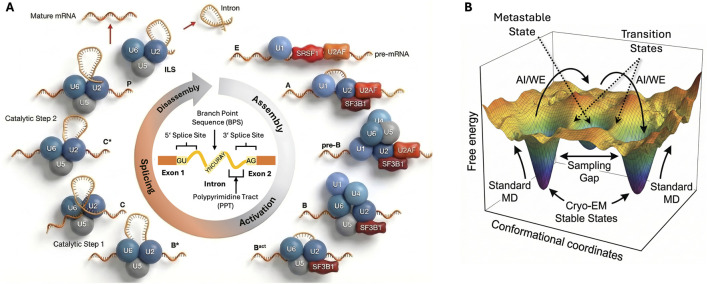
The spliceosome as a dynamic molecular machine. **(A)** Schematic of the canonical splicing cycle, highlighting stepwise assembly and extensive ATP-dependent remodeling. The key factors SF3B1 (A complex), U2AF1 (E complex), and SRSF2 (E/A complex) are indicated. The pre-mRNA shown in the center illustrates important elements for splice site recognition. Note that molecular shapes, sizes, and topology are stylized for visual clarity and do not represent structural data. **(B)** A free-energy landscape representation of biomolecular dynamics. While Cryo-EM captures structures in deep energy minima (stable states), biologically critical transitions require crossing high-energy barriers and accessing metastable intermediate states. The “sampling gap” illustrates the inability of standard MD to overcome these barriers on practical timescales, necessitating enhanced sampling methods (such as AI/WE) to bridge the gap and resolve the full conformational pathway.

Understanding the pathological deviations observed in cancer requires a general grasp of the healthy splicing cycle (Figure 1A). The spliceosome operates as a metallo-ribozyme: a catalytic RNA core coordinated by metal ions, encased within a protein scaffold that ensures fidelity (Fica et al., 2013). The splicing cycle is driven by ATP-dependent DExD/H-box RNA helicases, which power the conformational transitions required for assembly, activation, catalysis, and disassembly (Dörner and Hondele, 2024).

### 2.1 Early recognition and the E-Complex

Assembly initiates with the E complex, a cross-intron scaffold that establishes the molecular foundation for splice-site selection. U1 snRNP recognizes the 5′ splice site (5′SS), while the 3′ region is defined by the U2 auxiliary factor (U2AF) heterodimer. The large subunit, U2AF2, uses a flexible linker to bind the polypyrimidine tract (PPT), providing a modular anchor Sickmier et al. (2006). Concurrently, the small subunit U2AF1 secures the intron-exon boundary by specifically recognizing the conserved AG dinucleotide at the 3′ splice site (Wu et al., 1999).

### 2.2 Checkpoint control in the A-Complex

The transition to the A complex represents a decisive commitment step. Here, U2 snRNP base-pairs with the branch point sequence (BPS) to form the Branch Point Helix. This interaction bulges the branch adenosine, exposing it for future catalysis. The architecture is stabilized by the SF3b subcomplex, specifically SF3B1, which clamps around the helix using an induced-fit mechanism to position the nucleophile (Cretu et al., 2016). This stage acts as a critical fidelity checkpoint: the helicase DDX46 (Prp5) utilizes ATP hydrolysis to test the stability of the U2-BPS interaction, rejecting suboptimal sites via kinetic proofreading (Xu and Query, 2007).

### 2.3 Catalytic activation and intrinsic disorder

Subsequent recruitment of the U4/U6.U5 tri-snRNP triggers extensive remodeling by the helicase Brr2, which unwinds the U4/U6 duplex to release U6 for catalysis (Raghunathan and Guthrie, 1998). The resulting catalytic core functions as a metallo-ribozyme, mediating transesterification via RNA-coordinated divalent metal ions ( ${\text{Mg}}^{2+}$ ). However, final structural competence requires the DExD/H-box helicase Prp2 (DHX16). Prp2 destabilizes the SF3b subcomplex, effectively displacing it to expose the branch adenosine to the active site for the first reaction step (Ohrt et al., 2012; Wan et al., 2016).

Beyond these defined structures, the spliceosome relies on a high degree of intrinsic disorder. Proteins such as SRSF1 and the linkers between U2AF subunits utilize disordered regions to facilitate “fly-casting” mechanisms, effectively increasing the capture radius for molecular partners (Mackereth et al., 2011). While these fluid segments drive liquid-liquid phase separation (LLPS) and the formation of nuclear speckles (Kato et al., 2012), their conformational heterogeneity poses significant challenges for high-resolution modeling, representing a major frontier for structure-based drug design.

## 3 Pathogenic remodeling: how mutations rewire the spliceosome

Cancer-associated somatic mutations in the spliceosome are not random loss-of-function events but specific, positively selected lesions that reprogram the structural logic of RNA recognition (Yoshida et al., 2011; Dvinge et al., 2016). In the following, we will focus on recurrent mutations in core factors SF3B1, U2AF1, and SRSF2 to illustrate how discrete structural perturbations can drive transcriptome-wide oncogenic shifts, making them promising targets in therapeutic strategies.

### 3.1 SF3B1: the gatekeeper of branch point fidelity

**FIGURE 2:**
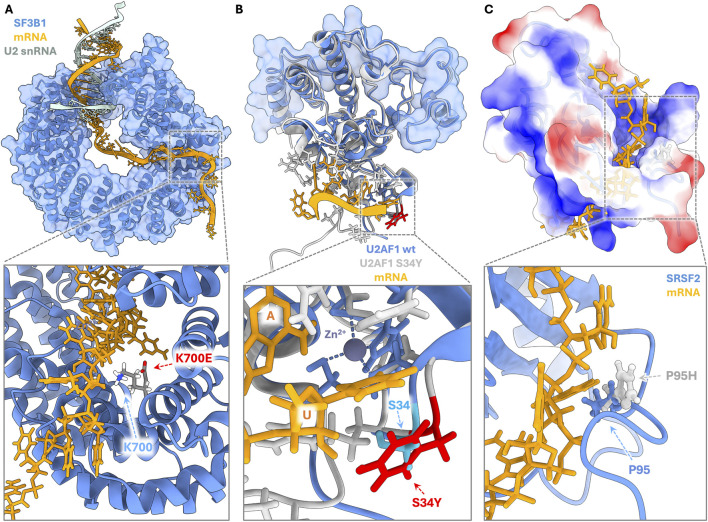
Atomic-level rewiring of RNA recognition by oncogenic mutations. **(A)** SF3B1 K700E. The heat-repeat domain of SF3B1 acts as a scaffold for the branch point sequence (BPS). The K700E substitution (modeled here on PDB ID 5Z56 (Zhang et al., 2018)) introduces a negative charge into the RNA-binding tunnel. This alters the local electrostatic environment, destabilizing the interaction with the canonical branch point adenosine. **(B)** U2AF1 S34F. An overlay of the wild-type U2AF1 zinc knuckle (blue and cyan, PDB ID 4YH8(Yoshida et al., 2015)) and the S34Y mutant complexed with RNA (grey and red, PDB ID 7C08 (Yoshida et al., 2020)). The S34Y mutation serves as a structural surrogate for the clinical S34F variant. The bulky aromatic side chain creates a steric clash with uridine at the −3 splice site position, driving a shift in specificity toward cytosine to relieve steric hindrance. **(C)** SRSF2 P95H. The Pro95 residue is critical for the orientation of the linker region relative to the RNA Recognition Motif (RRM) (PDB ID 2LEB (Daubner et al., 2012)). The P95H substitution alters the conformational equilibrium of the protein, changing its affinity to favor C-rich over G-rich Exonic Splicing Enhancer (ESE) motifs. Electrostatic surface potential illustrates the structural interface for mRNA recognition. Methodological Note: For panels (A) and (C), where specific mutant crystal structures were unavailable, mutant side chains were modeled using the Dunbrack rotamer library within UCSF ChimeraX (Meng et al., 2023).

The most frequent splicing lesions occur in *SF3B1*, with the K700E substitution in the HEAT repeat domain serving as the prototype in myelodysplastic syndromes and uveal melanoma (Papaemmanuil et al., 2011; Harbour et al., 2010). Structurally, the replacement of a positively charged lysine with a negatively charged glutamic acid disrupts the local electrostatics near the branch point helix (Cretu et al., 2016) (Figure 2A). Critically, this mutation uncouples SF3B1 from the cofactor SUGP1, which is normally required to recruit the proofreading helicase DHX15. The failure to recruit DHX15 effectively bypasses the kinetic proofreading checkpoint described above, allowing the spliceosome to engage aberrant, upstream branch points (Zhang et al., 2022; Xing et al., 2025). This geometric shift forces the selection of cryptic 3′ splice sites, generating pseudo-exonic inclusions in tumor suppressors such as *BRD9*, which subsequently undergo nonsense-mediated decay (Inoue et al., 2019). The detection of these cryptic 3′ splice sites in peripheral blood or bone marrow represents a potential high-sensitivity diagnostic biomarker for SF3B1-mutant clones, preceding clinical symptoms of hematologic transformation (Bak-Gordon and Manley, 2025; Dolatshad et al., 2016; Malcovati et al., 2020).

### 3.2 U2AF1: altering zinc finger specificity

Mutations in *U2AF1* fundamentally alter the recognition of the 3′ splice site AG dinucleotide through steric mechanisms (Yoshida et al., 2020; 2013). The recurrent S34F and S34Y mutations involve the substitution of a serine residue in the first zinc knuckle with a bulky aromatic side chain (phenylalanine or tyrosine, respectively). Structural analysis of the S34Y variant reveals that this substitution introduces a steric clash that reshapes the RNA-binding pocket, reducing the protein’s structural flexibility (Figure 2B). While the wild-type protein efficiently recognizes both pyrimidines at the −3 position (CAG or UAG), the mutant form exhibits a strict preference for cytosine while disfavoring uridine (Okeyo-Owuor et al., 2015; Ilagan et al., 2015). This altered specificity creates a transcriptome-wide filter: exons preceded by a cytosine are preferentially retained, while those with a uridine are skipped, driving the mis-splicing of oncogenes like *H2AFY* and *IRAK4* (Kim et al., 2021; Smith et al., 2019). Against this backdrop, *U2AF1* S34 mutations are now incorporated into molecular risk models as adverse prognostic biomarkers in myeloid neoplasms, and their associated splice isoform signatures (for example, *IRAK4*-L) are under active investigation as companion diagnostics for emerging IRAK4 and spliceosome-targeted therapies in precision oncology (Garcia-Manero et al., 2024; Badar et al., 2023; Li et al., 2020).

### 3.3 SRSF2: modulating exonic splicing enhancers

In contrast to intronic recognition defects discussed before, SRSF2 P95 hotspot mutations (primarily P95H, P95L, and P95R) remodel the recognition of exonic splicing enhancers (ESEs) (Daubner et al., 2012; Kim et al., 2015). P95 resides in the linker region between the RNA recognition motif (RRM) and the RS domain, where mutations induce conformational changes affecting the RRM termini (Figure 2C). This structural alteration shifts SRSF2’s RNA-binding specificity: whereas wild-type SRSF2 recognizes the degenerate SSNG motif (S=C or G) with similar affinity for both CCNG and GGNG variants, mutant SRSF2 exhibits enhanced binding to CCNG sequences and reduced affinity for GGNG motifs. This specificity switch drives widespread alterations in cassette exon inclusion and exclusion that can lead to nonsense-mediated decay, production of aberrant protein isoforms with altered function, and impaired hematopoietic differentiation (Liu et al., 2024; Rahman et al., 2020; Kim et al., 2015; Liang et al., 2018). At the clinical level, SRSF2 P95 mutations define a characteristic subset of myeloid neoplasms associated with adverse prognosis and are being incorporated into molecular risk stratification models (Hoff et al., 2024). Beyond prognostication, the distinct splicing signatures driven by these mutations are under active investigation as biomarkers for precision oncology, highlighting synthetic lethal vulnerabilities that currently remain in preclinical development (Su et al., 2024; Eldfors et al., 2024).

## 4 The sampling gap: challenges in simulating large-scale dynamics

Molecular dynamics (MD) has been an important tool for generating atomistic hypotheses about spliceosome structure-function relationships, refining cryo-EM models, probing local flexibility, and rationalizing the impact of mutations. Pokorná et al. provide an excellent review of the major MD studies on spliceosomal components (Pokorná et al., 2025). However, using only classical equilibrium MD as a primary engine for spliceosome drug discovery exposes several fundamental limitations, as the spliceosome pushes the boundaries of system size, timescale, and force-field accuracy (Baltrukevich and Bartos, 2023; Muscat et al., 2024; Migens et al., 2025).

The most fundamental constraint is the disparity between accessible simulation times and biological timescales (Figure 1B). Even on modern GPU clusters and specialized hardware, routine all-atom MD for systems the size of spliceosomal subcomplexes typically reaches microseconds at best. Achieving tens to hundreds of microseconds is possible but extremely costly, and millisecond trajectories remain exceptional rather than routine (Shaw et al., 2009; Gapsys et al., 2024). In contrast, functionally important spliceosomal transitions, such as the large-scale remodeling of snRNPs (e.g., B → B* activation), rearrangements of HEAT-repeat domains, or the opening of deep, transient pockets, occur on millisecond to second timescales. These motions often involve crossing substantial free-energy barriers and visiting sparsely populated intermediate states. This 3- to 6-order-of-magnitude gap means that straightforward equilibrium MD will overwhelmingly sample fluctuations near the starting structure (Hollingsworth and Dror, 2018).

Classical force fields such as AMBER and CHARMM have been extensively optimized for proteins and, more recently, substantially improved for RNA and protein-RNA complexes (Mlýnský et al., 2025). Nonetheless, spliceosomal systems present particularly stringent tests. RNA’s densely charged phosphate backbone and complex stacking/branching modes make its behavior highly sensitive to the treatment of long-range electrostatics, water models, and ion parameters. Residual inaccuracies can artificially weaken or over-stabilize RNA-protein interfaces, distort backbone conformations, or make folded RNA elements appear either too rigid or too labile compared to experiment (Mlýnský et al., 2025; Baltrukevich and Bartos, 2023; Šponer et al., 2018). Additionally, standard non-polarizable models fail to capture the complex coordination geometry and charge transfer of catalytic ${\text{Mg}}^{2+}$ and ${\text{K}}^{+}$ ions, often necessitating expensive QM/MM (Quantum Mechanics/Molecular Mechanics) approaches for accurate active site representation (Grotz et al., 2021; Šponer et al., 2018). And while explicit solvent is required to capture essential water-mediated interactions, its computational cost limits the exploration of rare transitions, a bottleneck that implicit solvent models cannot reliably address without distorting the delicate balance of protein-RNA forces (Chen et al., 2023). Sampling is further hindered by the rugged energy landscape. Because cryo-EM structures represent deep free-energy minima, unbiased MD rarely accesses higher-energy intermediates where allosteric pathways and cryptic pockets often reside, effectively inferring the plot of a movie from its opening frame (Vant et al., 2022; Verkhivker et al., 2023).

## 5 Enhanced sampling of rare events

To address the sampling bottlenecks of classical MD, a range of enhanced sampling strategies and statistical frameworks have been developed. These methods deliberately bias dynamics, restructure sampling protocols, or reduce system resolution to recover statistics on rare events and massive conformational changes standard MD cannot access. Reviews of enhanced sampling techniques for biomolecular simulations provide detailed guidance on methodology selection and implementation across diverse timescale regimes (Mehdi et al., 2024; Hooten et al., 2025; Yang et al., 2019). In the following, we focus on promising techniques for studying spliceosomal dynamics and cryptic binding site discovery on splicing factors.

### 5.1 Cryptic pockets and binding thermodynamics

Recent advances in identifying cryptic pockets and exploiting allosteric mechanisms have transformed the drug discovery landscape, enabling the targeting of previously inaccessible protein sites (Bemelmans et al., 2025; Mukhopadhyay et al., 2025). Mixed-solvent molecular dynamics (MSMD) simulations augment conventional MD by introducing small organic probes (e.g., benzene, isopropanol) that can identify and map these cryptic binding sites. (Mukhopadhyay and Chakrabarty, 2025; Vithani et al., 2024; Koseki et al., 2025; Chan et al., 2023; Wakefield et al., 2022; Smith and Carlson, 2021; Kuzmanic et al., 2020). These probes compete with water, clustering in thermodynamically favorable “hotspots” that reveal ligandable sites on flexible surfaces. Recent advances in topological data analysis combined with MSMD (Koseki et al., 2025) have significantly improved cryptic site prediction accuracy by assessing protein conformational variability during probe clustering, substantially outperforming recent machine-learning approaches. While MSMD identifies where ligands bind, alchemical methods such as Free Energy Perturbation (FEP) and Thermodynamic Integration (TI) quantify how strong that binding is (York, 2023). By physically transforming one ligand into another along a non-physical pathway, these methods calculate relative binding free energies with high accuracy (Ross et al., 2023; Sampson et al., 2024).

### 5.2 Kinetics and rare transition pathways

The Weighted Ensemble (WE) method accelerates rare events without biasing the underlying energy landscape (Chong and Zuckerman, 2025; Aristoff et al., 2023; Hellemann and Durrant, 2023). It partitions configuration space along a progress coordinate, splitting trajectories in under-sampled regions and merging them in over-sampled ones. Complementing WE, Markov State Models (MSMs) provide a framework to analyze aggregate simulation data (Konovalov et al., 2021; Bose et al., 2025; Liu et al., 2025). By discretizing high-dimensional conformational space into countable states and estimating transition probabilities between them, MSMs reconstruct long-timescale kinetics from many short, parallel MD trajectories. Recent application to RNA-ligand binding demonstrated MSM’s power for characterizing complex kinetic pathways in neomycin-riboswitch binding (Chyzy et al., 2024). For spliceosomal proteins, MSMs could similarly map the landscape of branch point adenosine and polypyrimidine tract recognition by SF3B1 and U2AF2, revealing metastable intermediates that may represent kinetic bottlenecks or allosteric vulnerabilities for therapeutic targeting.

### 5.3 Large-scale dynamics and sampling

The megadalton size of the spliceosome often precludes all-atom sampling of global rearrangements. Coarse-Grained (CG) MD models (e.g., Martini, G $\bar{\text{o}}$ -models) overcome this by grouping atoms into single interaction beads, reducing degrees of freedom and allowing larger time-steps (Souza et al., 2021; Yangaliev and Ozkan, 2025; Takada, 2019). CG is essential for simulating the massive domain reorganizations required during the spliceosome cycle, such as the transition from the B to the B^act^ complex, or the phase-separation behavior of intrinsically disordered regions in SR proteins (Zhan et al., 2024; Von Bülow et al., 2025).

For systems where atomistic detail is required but barriers are high, methods like Metadynamics apply history-dependent potentials to specific variables to force transitions (Laio and Parrinello, 2002). This forces the system out of local minima, allowing the calculation of free-energy landscapes for specific reaction coordinates, such as the bending of the SF3B1 superhelix or the mechanical opening of the branch point pocket (Rakesh et al., 2016; Zhan et al., 2024). Alternatively, Replica-exchange MD (REMD) and related schemes facilitate barrier crossing by running multiple simulations at different temperatures (or Hamiltonians) and periodically swap configurations (Zhu et al., 2025). While applying REMD to entire spliceosomal assemblies is typically prohibitive, targeted use on isolated domains or intrinsically disordered regions can improve exploration of alternative conformations.

## 6 From static structure to generative design

We are in the midst of a paradigm shift in structural biology driven by deep learning and generative AI (Malhotra et al., 2025). The field is moving beyond predicting a single “best” structure toward modeling thermodynamic ensembles, interfaces, and designing new biomolecules *de novo* (Cui et al., 2025; Schapira et al., 2024). Rather than explicitly integrating equations of motion as in Molecular Dynamics (MD), these models learn statistical regularities in sequence, structure, and interaction data. In favorable cases, they can sidestep the sampling bottlenecks that limit classical simulations, offering a new toolkit for targeting the spliceosome (Desai et al., 2024).

### 6.1 Foundation models for structural prediction

AlphaFold2 (AF2) established that protein structures could be predicted with near-experimental accuracy for many monomers (Jumper et al., 2021). Its successor, AlphaFold3 (AF3), extends this concept with a unified diffusion-based architecture capable of modeling protein-protein, protein-ligand, and protein-nucleic acid complexes within a single framework (Abramson et al., 2024). For spliceosomal biology, such models may provide a way to generate atomistic hypotheses for mutant splicing factors bound to RNA elements or small-molecule inhibitors that are experimentally challenging to crystallize.

In parallel, a growing ecosystem of open-weights foundation models has emerged, democratizing access to complex prediction. RoseTTAFold All-Atom (RFAA) pioneered the generalized modeling of protein-nucleic acid-small molecule complexes, offering an open-source alternative to proprietary systems (Krishna et al., 2024). Building on this, Boltz-2 represents a significant leap forward, predicting not only 3D structures of mixed-modality complexes (proteins, RNA, DNA, small molecules) but also binding affinities, achieving accuracy competitive with or superior to AF3 in recent benchmarks (Passaro et al., 2025; Xu et al., 2025). Similarly, CHai-1 offers a robust alternative for predicting protein-nucleic acid interfaces (Chai et al., 2024).

Despite their power, it is important to note that these models are not physics engines. They can “hallucinate” plausible-looking interactions for physically impossible contacts (e.g., steric clashes in novel drug scaffolds) or predict rigid structures for intrinsically disordered regions (IDRs) that should remain unstructured (Krokidis et al., 2025; Fang et al., 2025). Thus, the outputs should be treated as high-confidence hypotheses rather than ground truth (Schapira et al., 2024).

### 6.2 From single structures to dynamic ensembles

While powerful, the single-structure output of these recently developed foundation models reflects an inherent bias toward one thermodynamically stable conformation, usually the dominant state in experimental training data. However, the spliceosome is a heat-driven molecular machine that relies on distinct, often transient, conformations to function. Capturing this heterogeneity is essential for understanding mechanistic transitions, such as the destabilization of the ${\text{B}}^{\mathrm{act}}$ complex prior to catalysis or the conformational release of SF3B1, states that are often invisible to static crystallography but could be accessible to generative ensembles.

New algorithms are emerging as “generative MD surrogates,” sampling diverse conformations at a fraction of the computational cost of physical simulations (Cui et al., 2025; Aranganathan et al., 2025). BioEmu uses a diffusion-based framework to estimate equilibrium distributions directly from sequence, effectively capturing the Boltzmann distribution of states (Lewis et al., 2025). Similarly, AlphaFlow/ESMFlow adapts the AF2 architecture using flow matching to interpolate between noise and diverse structural states, providing a “pseudo-trajectory” of conformational flexibility (Jing et al., 2024). While these models sample equilibrium states orders of magnitude faster than MD, they forfeit temporal coherence, rendering them unable to predict transition kinetics or the causal pathways of conformational switching.

Complementary strategies steer existing predictors toward higher diversity. AF-Cluster systematically clusters or subsamples the Multiple Sequence Alignment (MSA) to reduce evolutionary constraints (Wayment-Steele et al., 2024; Sala et al., 2023). In contrast, Entropy Guidance (EGF) utilizes the full MSA but modifies AlphaFold2’s intermediate hidden states via an auxiliary loss function to disrupt the model’s bias (Wu and Feng, 2025). While these heuristics effectively identify alternative minima, they inherently lack thermodynamic calibration. AlphaFold2-RAVE (Vani et al., 2023) bridges this gap by using these diverse predictions to seed physics-based enhanced sampling, successfully recovering Boltzmann-weighted ensembles. However, strategies that rely on MSA subsampling (including AF-Cluster and RAVE’s initialization) remain limited by alignment depth, hindering application to rapidly evolving variants where evolutionary data is sparse.

### 6.3 Inverse design of binders and language models

Beyond understanding existing structures, generative AI is now tackling the “inverse design” problem: proposing sequences that fold into specific structures or programming RNA sequences to control splicing outcomes.

ProteinMPNN and LigandMPNN have become standard tools for sequence recovery, rapidly designing sequences for fixed backbones or small-molecule binders (Dauparas et al., 2022; 2025). However, the spliceosome requires targeting complex Ribonucleoprotein (RNP) interfaces. The recently introduced RFDpoly (RoseTTAFold Diffusion for Biopolymers) (Favor et al., 2025) addresses this gap, building on the RFdiffusion model to enable the simultaneous hallucination of nucleic acid backbones and protein binders (Watson et al., 2023). This allows for the *de novo* design of proteins that can lock specific RNA conformations, or synthetic RNAs that mimic U-snRNA structural motifs. A unique challenge in splicing is targeting Intrinsically Disordered Regions (IDRs), such as the RS domains of SR proteins. Here, generative approaches must be adapted to design peptide binders that remain flexible yet possess high affinity, decoupled from rigid folding constraints.

Newer pipelines like BindCraft integrate AlphaFold-Multimer weights with ProteinMPNN and physics-based filters to perform “one-shot” design of high-affinity binders Pacesa et al. (2025). This composite approach effectively targets challenging interfaces that often evade traditional docking. BoltzGen extends this frontier further, introducing a unified all-atom generative model that enables the co-design of binders for diverse modalities, including proteins, nucleic acids, and small molecules, with explicit structure-based reasoning (Stark et al., 2025). A key challenge are “adversarial” sequences, that have high-confidence structural prediction (e.g., high pLDDT or pAE scores) despite being biologically invalid, physically unstable, aggregation-prone, or unfolded in reality. This has made rigorous post-design filtration using methods like BindEnergyCraft for physics-based rescoring or orthogonal co-folding assays an essential step to separate viable candidates from these high-confidence false positives (Nori et al., 2025).

Parallel to structural design, Large Language Models (LLMs) are decoding the “grammar” of splicing regulation. While geometric models handle 3D coordinates, emerging RNA foundation models like RiNALMo (Penić et al., 2025), and ERNIE-RNA (Yin et al., 2025) leverage vast transcriptomic datasets to predict secondary structure and function directly from sequence, outperforming earlier models on unseen RNA families. For 3D design, gRNAde applies geometric deep learning to the inverse problem, conditioning on 3D backbones to generate sequences that adopt specific tertiary folds (Joshi et al., 2024). On the functional side, TrASPr (Transformer for Alternative Splicing Prediction) represents a specialized leap forward (Wu et al., 2025). Unlike general protein LLMs (e.g., ESM3), TrASPr is explicitly trained to predict tissue-specific splicing outcomes and can design RNA sequences to modulate inclusion levels of specific exons.

### 6.4 Validation of structural fidelity for reliable binder and biomarker prediction

As generative models for protein design and RNA processing proliferate, rigorous benchmarking is essential to distinguish genuine predictive power from hallucination. This validation is critical across all modalities discussed, from the *de novo* design of binders for disordered regions to the large language models (LLMs) predicting splicing outcomes. Recent benchmarking efforts have revealed critical limitations. Studies such as FoldBench and Runs N′ Poses have addressed the risk of data leakage, where structural redundancies between training and test sets inflate performance metrics (Xu et al., 2025; Škrinjar et al., 2025). By strictly segregating datasets based on sequence identity and structural similarity, these benchmarks assess true generalization capabilities. Results indicate that current performance relies heavily on memorization; accuracy declines sharply in out-of-distribution (OOD) scenarios, such as when models are tasked with predicting novel ligands or binding pockets dissimilar to those in the training set. Similarly, PoseBusters was developed to audit the physical viability of generated molecules by validating geometric accuracy and chemical plausibility, such as assessing bond lengths and flat angles. It demonstrated that even geometrically accurate models can produce chemically implausible structures with steric clashes (Buttenschoen et al., 2024), Furthermore, recent results from CASP16, a community experiment to advance methods of computing three-dimensional protein structure, underscored persistent challenges in complex prediction, particularly for antibody-antigen targets (Zhang et al., 2025). These limitations suggest that while AI can theoretically be used for spliceosome targeting or to predict events such as “splicing switch-points”, the structural transitions that lead to drug resistance, the current rate of hallucination and OOD failure poses a risk to both therapeutic design and biomarker discovery. Consequently, AI models function best as sophisticated filters to prioritize candidates for downstream Free Energy Perturbation (FEP) and wet-lab validation, ensuring that AI-predicted candidates are biophysically sound before they enter the precision oncology pipeline.

## 7 Translating structural dynamics into clinical strategy

### 7.1 Direct modulation of the spliceosome core

Pharmacological strategies have shifted from broad-spectrum modulation toward precision targeting of the spliceosome machinery and its auxiliary factors (Lv et al., 2025; Tseng and Obeng, 2024; Anczukow et al., 2024). First-generation modulators, such as pladienolide B and its analogs (E7107, H3B-8800), exploit the structural plasticity of the SF3B1 subunit. Cryo-EM and structural studies reveal that these agents bind within the branch point adenosine (BPA) binding tunnel, locking the complex in an open, pre-catalytic conformation. MD simulations suggest that this binding event reduces the internal cross-correlation of SF3B1 HEAT repeats, effectively freezing the functional plasticity required to accommodate the branch point adenosine (Amorello et al., 2025; Corrionero et al., 2011; Yokoi et al., 2011). While these agents bind wild-type and mutant SF3B1 with equal affinity, they induce preferential lethality in mutant cells by exploiting the reduced tolerance for additional splicing stress (functional selectivity) (Seiler et al., 2018; Wheeler et al., 2024). However, the lack of thermodynamic selectivity resulted in a narrow therapeutic index (Jiang et al., 2023). E7107 was discontinued due to ocular toxicity, and H3B-8800 failed to achieve objective responses in myelodysplastic syndrome, hampered by on-target dose-limiting toxicities such as QTc prolongation (Hong et al., 2014; Steensma et al., 2019).

### 7.2 Programmable targeting *via* antisense oligonucleotides

While small molecules modulate the protein machinery, antisense oligonucleotides (ASOs) offer a programmable logic to target the RNA substrate directly (Takeshima, 2025; Havens and Hastings, 2016). ASOs are chemically modified nucleic acids that base-pair with pre-mRNA to sterically block regulatory motifs with high sequence specificity. However, the therapeutic utility of ASOs has historically been limited by the dynamic nature of RNA folding; target sequences are frequently occluded within stable secondary structures, rendering them thermodynamically inaccessible. Here, the integration of generative AI could solve the scalability bottleneck. New deep learning frameworks can now predict dynamic RNA accessibility profiles and optimize ASO sequences to outcompete internal RNA base-pairing (Wu et al., 2025; Lin et al., 2024; Kang et al., 2025).

### 7.3 Allosteric modulation and cryptic pockets

An emerging approach that circumvents these shortcomings is to develop allosteric inhibitors targeting cryptic pockets unique to mutant conformations. Molecular dynamics simulations have successfully mapped dynamic drug binding landscapes, revealing transient, hydrophobic trenches between RNA recognition motifs (RRMs) in U2AF2 that are invisible to static crystallography (Borišek et al., 2020). Similarly, simulations have identified metastable pockets near the SF3B1 HEAT 5-7 repeats and the K700E mutation site, providing novel footholds for allosteric control (Rozza et al., 2023; Spinello et al., 2021). In contrast to pan-modulators, allosteric ligands aim to selectively bind the oncogenic variant, theoretically sparing essential wild-type splicing. Distinct from traditional inhibitors that block active sites, aryl sulfonamides function as “molecular glues,” acting as an interfacial wedge that reshapes the DCAF15 surface to create a high-affinity composite binding site for the α -helix 1 of the RBM39 RRM2 domain. Structural analysis confirms that the sulfonamide moiety mimics the side chain of an endogenous amino acid, allowing the complex to accommodate the RBM39 Gly268 residue, a site where any sterically larger mutations confer drug resistance (Han et al., 2017). This strategy causes a collapse in splicing efficiency and is currently under clinical evaluation (Bewersdorf et al., 2023).

### 7.4 Synthetic lethality

Chronic splicing stress creates downstream vulnerabilities in genome maintenance and protein homeostasis that can be exploited synthetically. Defective co-transcriptional splicing promotes R-loop accumulation and replication stress, rendering mutant cells critically dependent on ATR/Chk1 checkpoint signaling. Inhibitors of this axis show preferential toxicity in spliceosome-mutant models (Nguyen et al., 2018; Bland et al., 2023). This vulnerability extends to cells with cohesin mutations (e.g., *STAG2*), which lack the capacity to repair R-loop-induced damage, defining a convergent synthetic lethal axis (Wheeler et al., 2024). Transcriptome-wide mis-splicing burdens protein quality control systems. Beyond proteasome inhibition (Huang et al., 2020), recent studies highlight the Integrated Stress Response (ISR) as a key target. For example, *U2AF1*-driven mis-splicing sensitizes cells to ISR modulation (Jin et al., 2024). Furthermore, upstream snRNP biogenesis represents a critical bottleneck. PRMT5, which methylates Sm proteins to facilitate assembly, is essential for spliceosome-mutant cell survival. Inhibition of PRMT5 triggers a lethal collapse of snRNP availability, offering a distinct therapeutic avenue (Wu et al., 2024; Fong et al., 2019; Kohsaka et al., 2024). The clinical success of synthetic lethal approaches will depend on robust predictive biomarkers, such as defined splicing factor mutations and R-loop–associated replication-stress signatures, that can enrich for patients most likely to respond and thus operationalize precision oncology (Peng et al., 2025; Nguyen et al., 2018).

### 7.5 Splicing-derived neoantigens as immunotherapy targets

Beyond direct targeting of splicing factors, the aberrant transcripts they generate provide a second axis for therapy: tumor-specific neoantigens. By altering the choice of the splice site, splicing factor mutations create novel exon-exon junctions and retained introns that can be translated into peptide sequences that are not present in the normal human proteome (Li et al., 2024; Kwok et al., 2025). The total splicing neoantigen burden, quantified through the integration of transcriptomic data and structural modeling, is emerging as a potent prognostic biomarker. High-burden tumors often exhibit enhanced immunogenicity with shared, targetable neoantigens, correlating with improved survival and superior response rates to immune checkpoint blockade (ICB) therapies in treated cohorts (Li et al., 2024).

Most neoantigen studies have focused on peptides arising from single nucleotide variants (SNVs), which typically introduce single amino acid substitutions. In contrast, aberrant splicing often causes frameshifts, premature termination, or inclusion of intronic sequences, leading to longer stretches of novel amino acids or entirely new C-terminal tails (Kwok et al., 2025; Smart et al., 2018). Importantly, these splicing-derived neoantigens demonstrate substantially higher immunogenicity than SNV-derived peptides. Mass spectrometry immunopeptidome studies confirm that splicing neoantigens are naturally processed and presented on MHC class I molecules (Li et al., 2024; Kwok et al., 2025; Chai et al., 2022).

Because splicing factor mutations such as SF3B1^K700E^ and SRSF2 hotspot mutations recur across patients and influence splice-site choice in a reproducible manner, some resulting neojunctions are shared among individuals with the same mutation and compatible HLA types. Recent large-scale analyses have identified shared splicing neoantigens in up to 90% of melanoma patients (Li et al., 2024), and tumor-wide neojunctions from genes such as *GNAS* and *RPL22* that are spatially and temporally conserved across entire tumors, including at metastatic sites (Kwok et al., 2025). In myeloid neoplasms, the SF3B1^K700E^ mutation itself generates a neoantigen that has been validated as a CD8^+^ T cell target with nanomolar functional avidity (Biernacki et al., 2025). Such “public” neoantigens raise the possibility of partially off-the-shelf vaccine or T-cell receptor (TCR) therapies that target groups of patients rather than requiring fully individualized designs.

### 7.6 Computational discovery pipelines

Identifying splicing-derived neoantigens requires integrative pipelines that connect RNA processing to antigen presentation. Multiple specialized computational tools have been developed to systematically predict splicing neoantigens from RNA-seq data. SNAF (Splicing Neo Antigen Finder) combines splice junction calling with deep learning-based immunogenicity prediction (DeepImmuno), tumor specificity ranking (BayesTS), and splicing regulator identification (RNA-SPRINT) (Li et al., 2024). Other validated tools include SpliceMutr for pan-cancer analysis (Palmer et al., 2024), NeoSplice (Chai et al., 2022), and ASNEO for personalized alternative splicing-based neoantigen identification (Zhang et al., 2020). These workflows reconstruct altered open reading frames, enumerate candidate peptides, and filter against normal tissue expression to enrich for tumor-specific sequences.

Downstream modules predict MHC binding, proteasomal processing, and expression levels to prioritize candidates for experimental validation. However, computational predictions remain imperfect: mass spectrometry validation studies demonstrate that only a subset of predicted neoantigens are actually presented on HLA molecules, highlighting the need for orthogonal experimental confirmation including immunopeptidome profiling and T cell reactivity assays (Li et al., 2024; Chai et al., 2022; Huber et al., 2025; Tretter et al., 2023). Despite these limitations, scalable computational prioritization provides an essential first step to focus resources on the most promising splicing-derived candidates.

### 7.7 Structural immunoinformatics: predicting recognition

For a candidate peptide to be a useful target, it must be stably presented by MHC molecules and recognized by T cells. Sequence-based predictors such as NetMHCpan estimate peptide-HLA binding affinities by integrating both binding affinity and mass spectrometry-eluted ligand data (Reynisson et al., 2020). However, high predicted affinity alone does not guarantee immunogenicity. Recent advances in structural modeling of peptide-MHC (pMHC) complexes using fine-tuned AlphaFold models achieve sub-angstrom accuracy and can provide additional insight into peptide conformation, anchor residue placement, and the structural distinctiveness of neoantigen-pMHC surfaces from self-peptides (Motmaen et al., 2023; Mikhaylov and Levine, 2023; Glukhov et al., 2024). Crucially, static binding affinity does not always correlate with immunogenicity, and peptide–MHC complex stability can often be a better predictor of T-cell responses (Rasmussen et al., 2016). Molecular dynamics simulations use metrics such as peptide backbone root mean square fluctuation (RMSF, a measure of atomic positional fluctuation over time) to quantify dynamic stability within the MHC groove, which have been linked to differences in TCR recognition and functional responses (Ma et al., 2025; Tomasiak et al., 2022; Bell et al., 2021). In case studies of cancer neoantigens, single amino-acid substitutions, including at anchor positions, can reconfigure peptide–MHC allostery so that the neoantigen populates groove conformations that permissively gate TCR binding, even when MHC binding affinity is similar to the corresponding wild-type peptide (Custodio et al., 2023; Ma et al., 2025). Large-scale analysis of validated neoepitopes has identified the presence of hydrophobic and aromatic residues in the peptide binding core as among the most important features predicting T cell immunogenicity (Borch et al., 2024). In SF3B1-mutant uveal melanoma, splicing-driven shared neoantigens have been shown to elicit robust memory ${\text{CD}8}^{+}$ T-cell responses (Bigot et al., 2021). More recently, tumor-wide public neoantigens derived from recurrent splicing aberrations in genes such as *GNAS* and *RPL22* have been validated to trigger neoantigen-specific ${\text{CD}8}^{+}$ T-cell responses with nanomolar functional avidity and mediate antigen-dependent tumor cell killing (Kwok et al., 2025). These examples illustrate a path toward immunotherapies that exploit the distinctive neoantigen landscape created by splicing factor mutations.

The integration of these AI/MD-driven structural insights allows for a transition from qualitative neoantigen identification to quantitative clinical prediction. By moving beyond simple sequence enumeration to assess the structural “foreignness” and MHC-binding stability of the splicing-derived repertoire, we can derive a high-confidence “integrated neoantigen burden” score. This score serves as a critical prognostic biomarker in precision oncology, enabling the stratification of patients based on their likelihood of responding to ICB and providing a molecular rationale for the observed clinical variability in immunotherapy outcomes.

## 8 Conclusion

The spliceosome is increasingly shifting from a perceived “undruggable” black box of prohibitive complexity to a system with tractable, dynamic vulnerabilities, that serve as the foundation for novel diagnostic and prognostic frameworks. As discussed in this review, the combination of high-resolution structural biology, physics-based simulations, and emerging generative AI methods is beginning to narrow the gap between static atomic snapshots and the transient, fluid reality of RNA processing.

Somatic mutations in *SF3B1*, *U2AF1*, and *SRSF2* are now understood not simply as loss-of-function events, but as specific structural perturbations that rewire splicing decisions, conceptually reshaping the underlying energy landscapes to favor aberrant recognition events. Exploiting these defects will likely require the coordinated use of complementary computational approaches. Generative foundation models such as AlphaFold3 and Boltz-2 can, in favorable cases, rapidly propose structural hypotheses for complex ribonucleoprotein interfaces, while atomistic simulations and enhanced sampling methods remain essential for resolving “breathing” motions and cryptic pockets that are inaccessible to static models.

As generative AI continues to evolve, its specific architectures and tools will undoubtedly change. However, the broader shift towards using these models and simulations to analyse dynamic conformational ensembles rather than isolated structures is set to become a key theme in spliceosome-focused drug discovery.

Looking ahead, increasing structural and dynamic insight into mutant spliceosomes has the potential to support a range of therapeutic strategies. The mapping of mutation-specific conformational signatures has the potential to facilitate the design of context-sensitive small molecules that preferentially engage transient allosteric sites, or “molecular glues”, which could stabilise otherwise weak regulatory interactions within the splicing machinery. At the same time, the recurrent and partially predictable nature of some splicing alterations may reveal a pool of candidate tumour-specific neoantigens. As discussed, these may serve as high-value prognostic biomarkers, where the calculated ‘neoantigenic burden’, refined by AI-driven structural validation, could predict patient response to immunotherapy in a clinical setting.

Although substantial experimental, computational, and clinical challenges remain, the convergence of dynamic structural determination with AI-enabled design offers a realistic path towards multi-omics integration for precision oncology. This synergy has the potential to translate an increasingly detailed understanding of RNA processing into clinically actionable diagnostic and prognostic biomarkers and therapeutic strategies.
